# Social Factors Determine the Emergency Medical Admission Workload

**DOI:** 10.3390/jcm6060059

**Published:** 2017-06-09

**Authors:** Seán Cournane, Richard Conway, Declan Byrne, Deirdre O’Riordan, Seamus Coveney, Bernard Silke

**Affiliations:** 1 Department of Medical Physics and Bioengineering, St. James’s Hospital, Dublin 8, Ireland; sean.cournane@gmail.com; 2 Department of Internal Medicine, St. James’s Hospital, Dublin 8, Ireland; drrichardconway@gmail.com (R.C.); declangbyrne@gmail.com (D.B.); doriordan@stjames.ie (D.O.); 3 Envo-Geo Environmental Geoinformatics, Cork, Ireland; seamus@envogeo.ie

**Keywords:** deprivation, single-parent status, education, emergency medical admissions

## Abstract

We related social factors with the annual rate of emergency medical admissions using census small area statistics. All emergency medical admissions (70,543 episodes in 33,343 patients) within the catchment area of St. James’s Hospital, Dublin, were examined between 2002 and 2016. Deprivation Index, Single-Parent status, Educational level and Unemployment rates were regressed against admission rates. High deprivation areas had an approximately fourfold (Incidence Rate Ratio (IRR) 4.0 (3.96, 4.12)) increase in annual admission rate incidence/1000 population from Quintile 1(Q1), from 9.2/1000 (95% Confidence Interval (CI): 9.0, 9.4) to Q5 37.3 (37.0, 37.5)). Single-Parent families comprised 40.6% of households (95% CI: 32.4, 49.7); small areas with more Single Parents had a higher admission rate-IRR (Q1 vs. for Q5) of 2.92 (95% CI: 2.83, 3.01). The admission incidence rate was higher for Single-Parent status (IRR 1.50 (95% CI: 1.46, 1.52)) where the educational completion level was limited to primary level (Incidence Rate Ratio 1.45 (95% CI: 1.43, 1.47)). Small areas with higher educational quintiles predicted lower Admission Rates (IRR 0.85 (95% CI: 0.84, 0.86)). Social factors strongly predict the annual incidence rate of emergency medical admissions.

## 1. Introduction

Deprivation [1] is associated with a marked increase in hospital admission rates [2], increased length of stay [3] and has been shown to influence mortality [4,5]. Although organizational reform has improved mortality outcomes following an acute emergency medical admission, there is an undoubted high acute [6,7] and 1-year mortality rate [8]. Importantly, the emergency medical admission rate being a non-discretionary event can be proposed as a healthcare outcome measure to examine the impact of social or environmental factors on health in the broadest sense.

A Deprivation Index may be derived, from methodology similar to that of Townsend [1] and Carstairs [9], producing an Index based on four indicators, relating to unemployment, social class, type of housing tenure and car ownership [10]. One of society’s most vulnerable groups, however, is Lone or Single Parents [11], while adverse effects on education and health for offspring of single-parent families have been attributed to material disadvantage [12]. The literature offers opposing views on the longer-term physical health of single- versus two-parent family offspring [13,14]. Furthermore, higher educational levels lead to health literacy with a range of cognitive and social skills which enable adults to use information to promote good health [15]. The interaction of deprivation status, single-parent status, the level of educational attainment and the emergency admission is of interest given that the catchment area of our hospital is predominantly located in the inner city, combining over 50% of patients labeled as deprived [16]. We have previously investigated the effect of deprivation on elderly patients; however, the relationship between admission incidence and the aforementioned social factors have not been investigated [17]. Accordingly, in this paper, using our database of all emergency medical admissions from the St James’s Hospital (SJH) catchment area admitted between 2002 and 2016, we investigate whether the emergency admission incidence rate is a sensitive indicator of healthcare outcomes as related to local area social factors.

## 2. Experimental Section

### 2.1. Background

St. James’s Hospital, Dublin, serves as a secondary care centre for emergency admissions in a catchment area with a population of 270,000 adults. All emergency medical admissions (70,543 episodes in 33,343 patients) within the catchment area of St. James’s Hospital, Dublin, were examined between 2002 and 2016, the operation and outcome of which have been described elsewhere [7,18].

### 2.2. Data Collection

An anonymous patient database was employed, assembling core information from each clinical episode including details from the patient administration system, national hospital in-patient enquiry (HIPE) scheme, the patient electronic record and laboratory data. HIPE is a national database of coded discharge summaries from acute public hospitals in Ireland [19,20]. The International Classification of Diseases, Ninth Revision, Clinical Modification (ICD-9-CM) has been used for both diagnosis and procedure coding from 1990 to 2005, and ICD-10-CM since then. Data included parameters such as the unique hospital number, admitting consultant, date of birth, gender, area of residence, principal and up to nine additional secondary diagnoses, principal and up to nine additional secondary procedures, and admission and discharge dates. Additional information cross-linked and automatically uploaded to the database includes physiological, haematological and biochemical parameters.

### 2.3. Deprivation Indices

Census return reports are based on the Electoral Divisions (EDs), the smallest administrative areas for which population statistics are reported. Using principle components analysis (PCA), a weighted combination of four indicators, relating to unemployment, social class, type of housing tenure and car ownership, was derived, as described by the Small Area Health Research Unit (SAHRU) investigators [10]. The Deprivation Index Scores were ranked from low (least deprived) to high (most deprived) and divided into quintiles or deciles according to their ranked raw scores, as described previously [21]. We utilized the registered address on our Patient Administration System to allocate each address to a divisional area, with a corresponding matched SAHRU Deprivation raw score and decile rank. These attribute data were joined to the small area polygon geometries based upon their relative geographic positions, using the ArcGS Geographic Information System (GIS) software implementation of the Point-in-Polygon algorithm [22].

From the 2006 Census returns information for each ED, the single-parent frequency and highest education level achieved for each ED were calculated as the ratio to the population within each ED. The dependency ratio was also calculated as the proportion of non-working (<15 or ≥65 years) relative to the total population in each census small area. We then used the quintiles of these values across the 74-hospital catchment area EDs as a regressor against the hospital admission rate incidence. The latter for emergency medical admissions was calculated by summing admissions from each ED over 15 years and calculating an average rate for each (numerator)/1000 local area population (denominator).

### 2.4. Risk Predictors

Derangement of admission biochemical parameters may be utilised to predict clinical outcome. We derived and applied an Acute Illness Severity score [23], predicting in-hospital mortality from the following parameters recorded in the Emergency Department [24]. A weighted age-adjusted score was derived; six risk groups (I–VI) were identified with cut-points for 30-day in-hospital mortality set at 1, 2, 4, 8 and 16%. We adjusted for Co-Morbidity using the Charlson Index [25] and disabling disease [26] IDC9/ICD10 discharge codes of 1, 2, 3 or 4 separate systems (e.g., cardiovascular, respiratory, diabetes, renal). In addition, sepsis categories of (1) No Culture requested (2) Culture Negative and (3) Culture Positive were examined.

### 2.5. Statistical Methods

Descriptive statistics were calculated for background demographic data, including means/standard deviations (SD), medians/inter-quartile ranges (IQR), or percentages. Comparisons between categorical variables and mortality were made using chi-square tests.

For hospital Admission Rates, we employed a truncated Poisson regression model, including predictive outcome categorical variables (e.g., disabling score groups) in the model as a series of indicator variables. The dependent variable of the Admission Rate is restricted to certain values; the predictor variables are therefore regressed against Admission Rates using the truncated Poisson model. We used robust standard errors for the parameter estimates, as recommended by Cameron and Trivedi [27]. The Poisson regression coefficients are the log of the rate ratio: the rate at which events occur is called the incidence rate. Thus, with the truncated Poisson regression model, we can interpret the coefficients in terms of Incidence Rate Ratios (IRR). We used the margins command in Stata v.13.1 (Stata Corporation, College Station, TX, USA) to estimate and interpret adjusted predictions for sub-groups, while controlling for other variables such as illness severity, using computations of average marginal effects. Margins are statistics calculated from predictions of a previously fitted model at fixed values of some covariates and averaging or otherwise over the remaining covariates. In the multi-variable model (logistic or Poisson), we adjusted univariate estimates of effect, using the previously described outcome predictor variables. The model parameters were stored; post-estimation intra-model and cross-model hypotheses could thereby be tested.

Adjusted odds ratios (OR) and 95% Confidence Intervals (CI) or IRRs were calculated for those predictors that significantly entered the model (*p* < 0.10). Statistical significance at *p* < 0.05 was assumed throughout. Stata v.13.1 statistical software was used for analysis.

## 3. Results

### 3.1. Patient Demographics

A total of 96,305 episodes in 50,612 unique patients were admitted as medical emergencies from the hospital catchment area over the 15-year study period (2002–2016). These episodes included all emergency medical admissions, including patients admitted directly into the Intensive Care Unit (ICU) or High Dependency Unit (HDU), respectively. The proportion of males was 48.7%. The median (IQR) length of stay (LOS) was 5.2 (2.0, 13.1) days. The median (IQR) age was 62.1 (40.3, 78.4) years, with the upper 10% boundary at 86.1 years. Patients who were resident within the catchment area were 33,343 with 70,543 of the total episodes. The latter cohort was used for admission rate incidence calculations.

### 3.2. Emergency Medical Patient Admission Characteristics

There were a total of 74 EDs in the catchment area with a recorded population of 210,443 persons in 2006. The median population per ED was 2845 (IQR 2020, 3399). These areas have a high element of Deprivation, being ranked nationally as Deprivation Quintile I (*n* = 13), Quintile III (*n* = 5), Quintile IV (*n* = 4) and Quintile V (*n* = 49) (Table 1).

The demographic characteristics (Table 1) of the admission profile by the Deprivation status (Quintiles I/III vs. IV/V i.e., lower or higher areas of disadvantage) are of interest; this is tabulated by Acute Illness Severity [23,28], Charlson Co-Morbidity Index [25], Chronic Disabling Disease Score [26] and Major Disease Primary Codes in the Respiratory (MDC4), Cardiovascular (MDC5) or Neurological (MDC1). Sepsis status, an objective parameter, is based on whether a patient had a blood culture or not and whether the culture was positive or negative. In this work, Sepsis was related to the 30-day in-hospital mortality rate. The respective overall mortalities by episode were no culture 2.4%, culture done but negative 9.4% and culture done with growth 13.7%. We have included this information in the results.

Patients from the more affluent EDs within the catchment area were admitted on average at a much older age, 74.9 years (95% CI: 58.3, 83.2) compared with those from the more disadvantaged areas, 64.3 years (95% CI: 45.1, 77.9). They were more likely to be female (54.6%). Patients from deprived areas had considerably higher admission rate incidence—33.9 (25.8, 41.8)/1000 population/per annum versus the more affluent areas 14.0 (7.7, 16.2). Single-Parent families on average comprised 40.6% of households (95% CI: 32.4, 49.7); this status varied markedly from the more advantaged Q1 20.4% (95% CI: 13.1, 25.8) to disadvantaged areas Q5 65.0% (95% CI: 58.4, 66.9).

### 3.3. Deprivation Parameters and Admission Incidence Rates

**Figure 1:**
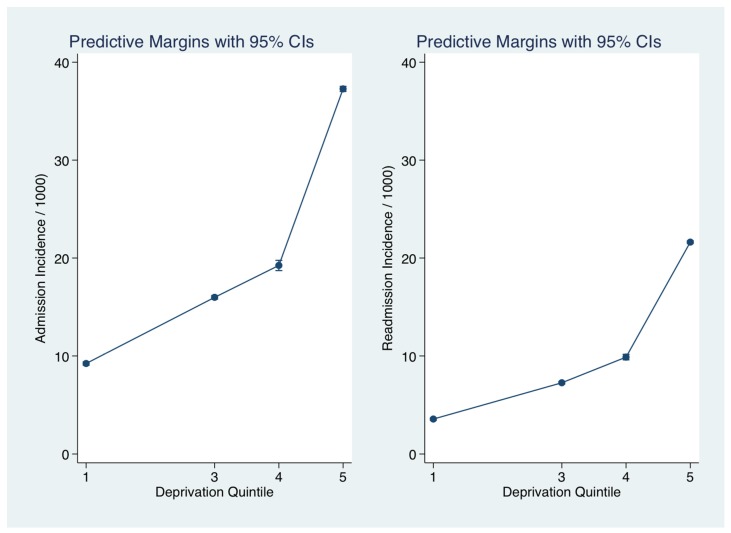
Relationship between Deprivation status and Hospital Admission Incidence rates for emergency medical conditions. The predicted probabilities for each were derived from the zero-truncated Poisson model. We used margins to estimate the average marginal effect. Graphed is Admission Rates/1000 local area population between 2002–2016 for the first or any subsequent admission.

**Figure 2:**
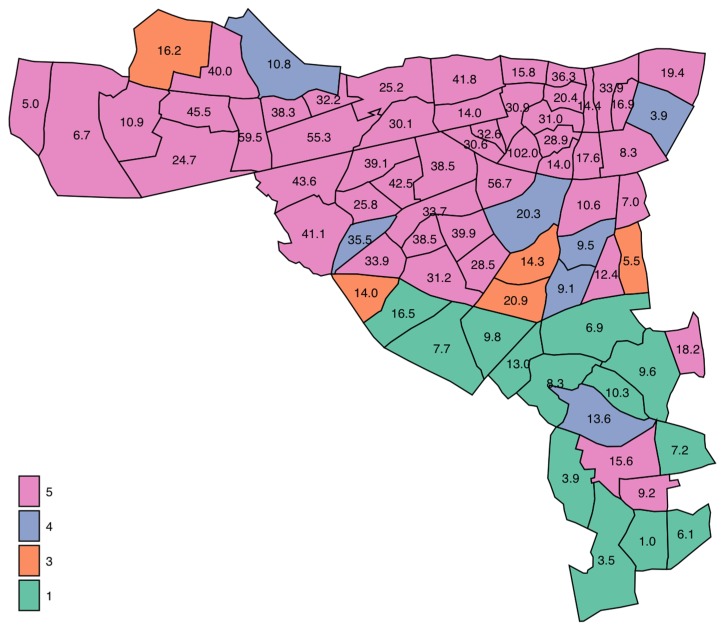
Spatial variation in Emergency Hospital Admission Incidence rates for medical conditions. There was a total of 74 EDs in the catchment area with a median population per ED of 2845 (IQR 2020, 3399). These areas have a high element of Deprivation, being ranked nationally as Deprivation Quintile I (*n* = 13), Quintile III (*n* = 5), Quintile IV (*n* = 4) and Quintile V (*n* = 49). ED = Electoral Division.

The relationship between deprivation status and hospital admission incidence rate for medical emergencies was calculated after adjustment for other significant predictors including Acute Illness Severity [23,28], Charlson Co-Morbidity Index [25], Chronic Disabling Disease Score [26] and sepsis status [29]. The adjusted admission rate incidence (Figure 1) increased approximately fourfold (IRR 4.0 (3.96, 4.12)) with Deprivation Quintile from Q1 from 9.2/1000 (95% CI: 9.0, 9.4) to Q5 37.3 (37.0, 37.5). There was a heavy concentration from the inner city with the upper 90 and 95 centile rates were 41.8 and 55.3/1000 respectively (Figure 2). The age at admission progressively fell with Deprivation status; compared with Q1, aged on average 69.0 years (95% CI: 68.6, 69.5), the median ages for Q4 and Q5 were 65.4 years (64.8, 65.9) and 60.8 years (95% CI: 60.7, 61.0), respectively.

### 3.4. Other Small Area Descriptors and the Admission Incidence Rates

**Figure 3:**
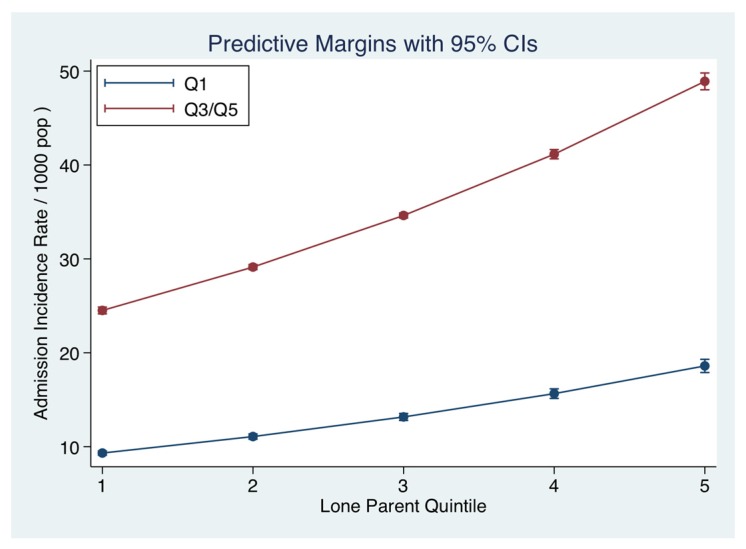
Relationship between Single-Parent status and Admission Incidence Rate of an emergency medical admission. The predicted probabilities were derived from the multi-variable truncated Poisson model. We used margins to estimate and interpret adjusted predictions for sub-groups, while controlling for other variables, using computations of average marginal effects. Single-Parent small area frequency predicted an increased admission rate, adjusted for Deprivation status.

The relationship between the Single-Parent status (related to Quintiles from low to high) and the hospital admission incidence for medical emergencies was calculated after adjustment for other significant predictors including Deprivation status. Single-Parent families on average comprised 40.6% of households (95% CI: 32.4, 49.7); this status varied markedly from the more advantaged Q1 20.4% (95% CI: 13.1, 25.8) to disadvantaged areas Q5 65.0% (95% CI: 58.4, 66.9). Adjusted for Deprivation status, the annual admission incidence rates/1000 population (Figure 3) across the Single-Parent Quintiles increased as follows from Q1 22.5 (95% CI: 22.2, 22.8) to Q5 50.0 (95% CI: 49.1, 50.9). The IRR was increased with an overall admission IRR (compared with Q1) for Q5 of 2.92 (95% CI: 2.83, 3.01).

**Figure 4:**
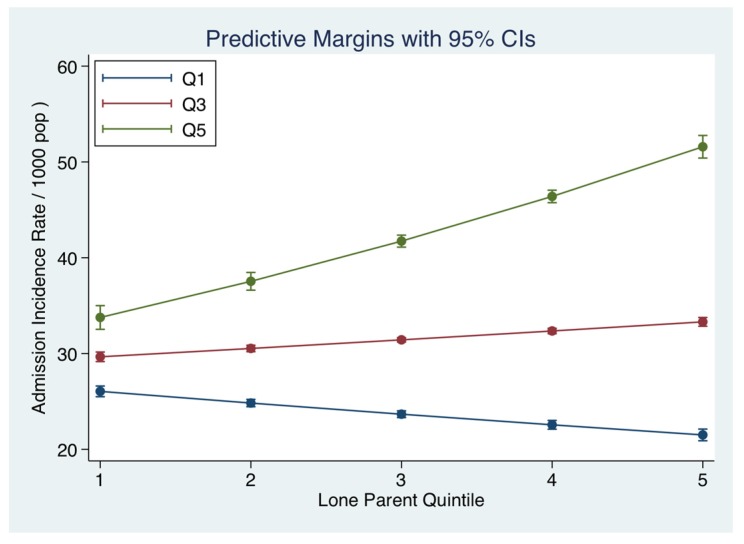
Interaction between small area Single Parent and unemployment rates and the Admission Incidence Rate of an emergency medical admission. The predicted probabilities were derived from the multi-variable truncated Poisson model. We used margins to estimate and interpret adjusted predictions for sub-groups, while controlling for other variables, using computations of average marginal effects. As the Quintiles (Q1, Q3, Q3) of unemployment increased, the strength of the association of Single-Parent status with the admission rate increased.

The admission incidence rate was further examined comparing educational levels. An education completion level to primary (<15 years), secondary (<18 years) or tertiary level (>18 years) could influence or interact with Single-Parent status. The frequency data for Education was as follows (median with 95% CI): <15 years 12.8% (7.2, 19.5%), >15–18 years 20.6% (13.2%, 23.7%) and >18 years 24.5% (17.6%, 33.3%). The admission incidence rate was higher for Single-Parent status (IRR 1.50 (95% CI: 1.46, 1.52)) with the small area frequency of educational completion at primary level also being a signifant predictor (IRR 1.45 (95% CI: 1.43, 1.47)) (Figure 4). However, for small areas with a higher frequency of educational completion level >18 years, the Single-Parent status was not as strong a predictor of the admission incidence rate (IRR 1.05 (95% CI: 1.04, 1.06)) and small areas with more higher education predicted lower Admission Rates (IRR 0.85 (95% CI: 0.84, 0.86)).

### 3.5. Multivariable Model with Adjustment for Deprivation Index

In the full multivariate model, we included the Single-Parent status, the Electoral Division Dependency Ratio (proportion of population <15 or ≥65 years within each ED), the National Deprivation Index (3440 Electoral Divisions ranked nationally by decile), with adjustment for Acute Illness Severity, Charlson Co-Morbidity Index, Chronic Disabling Disease Score and Sepsis status. The Single-Parent status, age at which education was completed (Educational level), and the population Dependency Ratio together with the National Deprivation Index were each independently predictive of the annual incidence rates of an emergency medical admission (Table 2).

## 4. Discussion

This study has shown that in our society there are communities living in adjacent small areas with quite different healthcare outcomes; the variation in the annual emergency admission rate incidence from these groups can vary eightfold from 5/1000 to 40/1000 [30,31,32] and where there are many dependents (i.e., proportions of young <15 years and older persons ≥65 years) within such areas, the annual Admission Rates can rise further to 60–80/1000 [30,31]. While our data relates to one hospital only and uses a different albeit related Deprivation Index methodology, the absolute admission incidence rate appears quite similar to those calculated for UK data [33]. The acute 5% [6,7] and 1-year mortality rates of 20–40% [8,34] suggest that an emergency medical admission is a sentinel life event with consequence. From our study, the patients from the more deprived areas present nearly a decade earlier than patients from more affluent areas.

Within our catchment area, the Deprivation Index was a major predictor of the hospital emergency admission rate incidence rate; however, there are other Small Area Population Statistics (SAPS) not taken into account in the calculation of Deprivation status. The admission incidence rates were markedly increased (2.36 (95% CI: 2.28, 2.45)) from small areas with more single-parent families. Indeed, single parents represent a vulnerable group in society with two out of every three single parents reportedly living in poverty [11]. When considering health outcomes in a community as related to single-parent family status, it is necessary to consider the effect both on the child and the parent. Single parenthood can result in more anti-social behaviour in children [12], and lower levels of educational achievement when compared with children of two-parent families [34]. Similarly from a health outcome perspective, single-parent families prove to be at a disadvantage with the association between one-parent families and child mortality attributable to their lower socio-economic position [35]. Almost counter intuitively, the difference between children from intact and non-intact families is reportedly small over the long-term, with offspring of single-parent families mostly in as good physical health as those from two-parent families [13,14]. We can only, of course, consider the health impact for the adults admitted via our emergency system. Our analysis does not attempt to disentangle the long-term influence on single-parent children; however, our data suggests that with an increasing prevalence of single-parent families in society [13], there may be a greater number of emergency medical admissions.

Our data suggested that educational progression to a tertiary level mitigated some of the adverse impact that, broadly, might otherwise have been anticipated; small areas with higher proportions of educated household tended to have better healthcare outcomes, in terms of a lower rate of emergency hospital emergency admissions. Indeed, an emerging theme in healthcare is the concept of health literacy relating educational level and consequence skills in ability to access, utilize and empower healthcare decisions. In general, this is defined as the capacity to obtain, process, and understand basic health information and services needed to make appropriate health decisions. Broader definitions focus on a wider range of cognitive and social skills which enable adults to use information to promote good health [15]. How health literacy affects health status and health service utilization remains unclear [36]; however, it could be argued that core education level and achievements may be crucial. Education can also be shown to exert an effect on life expectancy; a 35-year-old man educated to primary level will live on average for another 41.3 years, this figure would rise to 44.5 years for those with a secondary school education and to 46.9 for those with a third level education. The corresponding figure for a 35-year-old woman was 45.6, 48.5 and 50.4 years [37].

The hospital studied in this work serves a catchment area of much poverty [16], with 47 of the 74 Electoral Divisions categorised in the top quintile of the National Deprivation Index [10]. While this work was not centred on poverty and its healthcare consequences, the social deprivation may equate to a lack of access to a level of income that would allow an individual or family to meet a prescribed set of basic needs. The usefulness of this concept of poverty as a focus of analysis for social justice has been questioned, with the suggestion that the impairment of the potential of the individual and deprivation is limiting a capacity to develop and maximise that potential [38]. The inferences that can be drawn from the strong associations of social factors and the emergency hospital incidence rate are, of their nature, generalised and cannot be very specific. When considering the effect of social circumstances on community health and the adverse health effects, our approach has been to emphasise deprivation as the result of community rather than personal disadvantage. The census-derived parameters have been applied to small areas with the differences across areas, then used as predictors of the rates of emergency admissions. It is therefore considering community disadvantage in aggregate and indicates the importance of area of residence influencing the healthcare outcomes. Outcomes associated with residence in areas designated as deprived by census-based indicators are reportedly wholly explained by the concentration in those areas of people with adverse personal or socio-economic factors [39].

As with any, this one has its limitations. We have included a large emergency medical admission cohort, encompassing 15 years of episodes and reflecting real-world clinical practice; however, the work has been conducted in one institution, is local and, as a result, its findings may not be generalised to the population as a whole. There are groups of patients in more affluent areas who may present as emergencies outside the public health system, unlike the more deprived who have little other recourse when an emergency occurs. These patients will not be captured by our statistics and thus there may be an underrepresentation of the admission incidence rates of patients from the more affluent areas. In addition, the study only included emergency medical admissions and did not include all admissions to the emergency department and, thus, the admission rate and proportion of emergency department patients that were admitted were not presented. The Charlson Comorbidity Index Score was computed using diagnoses during the index admission only and, thus, the comorbidity level may have been underestimated by this. Furthermore, we were unable to include lifestyle factors, such as smoking and alcohol consumption, which may present as a limitation. Further, our ED populations come from the 2006 Census, although there may have been some variation over the study period.

## 5. Conclusions

To conclude, the current study shows that in addition to the overall National Deprivation Index community, there are other census parameters that may be important predictors of the emergency medical admission rate incidence. When adjusted for illness severity, family structure, final educational level in addition to deprivation status were all independent predictors of admission incidence. Thus, this study highlights the importance of social factors on healthcare outcomes and the need to address deprivation in society.

## Figures and Tables

**Table 1:** Emergency Admissions (2002–2016): Demographics by Deprivation Status.

Factor	Level	Quintiles I/III	Quintiles IV/V	*p*-Value
*N*		6150	56,219	
Gender	Male	2791 (45.4%)	27,654 (49.2%)	<0.001
	Female	3359 (54.6%)	28,565 (50.8%)	
Outcome	Alive	5750 (93.5%)	53,479 (95.1%)	<0.001
	Died	400 (6.5%)	2740 (4.9%)	
Age, median (IQR)		74.9 (58.3, 83.2)	64.3 (45.1, 77.9)	<0.001
Length of Stay (days)		5.5 (2.4, 10.8)	5.3 (2.3, 10.0)	<0.001
Admission Rate *		14.0 (7.7, 16.2)	33.9 (25.8, 41.8)	<0.001
Readmissions		1.0 (0.0, 2.0)	2.0 (0.0, 4.0)	<0.001
Acute Illness Severity	1	117 (2.1%)	1373 (2.7%)	<0.001
	2	261 (4.7%)	3305 (6.4%)	
	3	459 (8.2%)	5973 (11.6%)	
	4	726 (13.0%)	8637 (16.8%)	
	5	1156 (20.7%)	10,454 (20.3%)	
	6	2863 (51.3%)	21,748 (42.2%)	
Charlson Index	0	2788 (45.4%)	23,704 (42.3%)	<0.001
	1	1658 (27.0%)	16,666 (29.7%)	
	2	1693 (27.6%)	15,730 (28.0%)	
Disabling Disease	0	578 (9.4%)	5696 (10.1%)	0.014
	1	1432 (23.3%)	13,666 (24.3%)	
	2	1771 (28.8%)	16,269 (28.9%)	
	3	1390 (22.6%)	12,359 (22.0%)	
	4	979 (15.9%)	8229 (14.6%)	
Sepsis Group	1	4757 (77.3%)	42,985 (76.5%)	0.073
	2	1171 (19.0%)	11,347 (20.2%)	
	3	222 (3.6%)	1887 (3.4%)	
MDC Respiratory		4796 (78.0%)	40,554 (72.1%)	<0.001
		1354 (22.0%)	15,665 (27.9%)	
MDC Cardiovascular	0	4906 (79.8%)	46,879 (83.4%)	<0.001
	1	1244 (20.2%)	9340 (16.6%)	
MDC Neurology	0	5015 (81.5%)	47,296 (84.1%)	<0.001
	1	1135 (18.5%)	8923 (15.9%)	

Note: LOS: length of stay; MDC: Major disease category; IQR: Inter-Quartile Range. * Calculated for patients resident in catchment area.

**Table 2:** Truncated Poisson regression model to predict Hospital Admission Rates.

Predictor	IRR	SE	*z*	*p* > *z*	95% CI	
Single Parent						
Q2	1.18	0.02	10.4	0.000	1.14	1.21
Q3	1.45	0.02	25.6	0.000	1.41	1.49
Q4	1.33	0.02	21.0	0.000	1.30	1.37
Q5	1.69	0.02	30.5	0.000	1.63	1.75
Education <15 years						
Q2	1.46	0.03	16.9	0.000	1.40	1.53
Q3	1.22	0.03	9.1	0.000	1.17	1.27
Q4	1.07	0.02	3.3	0.001	1.03	1.12
Q5	1.19	0.02	8.8	0.000	1.15	1.24
Dependency Ratio						
Q2	1.11	0.02	6.5	0.000	1.07	1.14
Q3	1.97	0.03	44.4	0.000	1.91	2.03
Q4	1.56	0.02	30.6	0.000	1.51	1.60
Q5	1.82	0.02	36.8	0.000	1.76	1.88
Deprivation Index						
Q3	1.54	0.04	17.8	0.000	1.47	1.62
Q4	2.13	0.05	33.0	0.000	2.04	2.23
Q5	2.94	0.05	62.2	0.000	2.84	3.04

Note: IRR—the Incidence Rate Ratios for the Poisson model—these are derived by exponentiating the Poisson regression coefficient. There are two interpretations of the calculations: (1) the log of the ratio of expected counts explaining the “ratio” and (2) counts as number of events per time (or space), hence “rate” in Incidence Rate Ratio. CI: Confidence Interval; SE: Standard Error.
